# The paradigm shift in neural stem cells basic research driven by artificial intelligence related technologies

**DOI:** 10.3389/fncel.2025.1696943

**Published:** 2025-11-21

**Authors:** Pengfei Li, Yuehua Li, Chunfang Wang

**Affiliations:** 1 Translational Medicine Research Center, Shanxi Medical University, Taiyuan, Shanxi, China; 2 College of Computer Science and Technology (College of Data Science), Taiyuan University of Technology, Taiyuan, Shanxi, China; 3 Laboratory Animal Center, Shanxi Medical University, Taiyuan, Shanxi, China

**Keywords:** neural stem cells, artificial intelligence, deep learning, machine learning, computational neuroscience

## Abstract

Neural stem cells (NSCs) hold significant potential in neural regenerative medicine, yet research faces multiple challenges such as cellular heterogeneity, unclear microenvironment interactions, and low clinical translation efficiency. In recent years, the rapid development of artificial intelligence (AI) technologies has provided new ideas and tools to address these issues. This paper reviews the current applications of AI in fundamental NSCs research, including intelligent identification, deep learning-driven subtype analysis, spatial microenvironment deconstruction, and dynamic analysis of neural differentiation. Additionally, we discuss several key AI technologies not yet applied to NSCs research, such as generative adversarial networks, graph neural networks, and self-supervised learning, as well as their potential applications in cell classification, interaction network analysis, and morphological feature extraction. Although AI technologies show great promise in NSCs research, challenges remain regarding data quality, model robustness, and interpretability. Therefore, future research should focus on establishing high-quality standardized multimodal data platforms and integrating biological knowledge to enhance model interpretability, thereby deepening the understanding of NSCs biological characteristics and differentiation mechanisms and advancing personalized therapies.

## 1 Introduction

Neural stem cells (NSCs) are a type of multipotent cells present in both the embryonic and adult central nervous system, possessing self-renewal capacity and the ability to differentiate into neurons, astrocytes, and oligodendrocytes (Kriegstein and Alvarez-Buylla, 2009; Nam et al., 2015; Reynolds and Weiss, 1992). They demonstrate revolutionary potential in neural regenerative medicine, capable of repairing neuronal loss in neurodegenerative diseases such as Parkinson’s and Alzheimer’s diseases through transplantation (Deokate et al., 2024; Olanow et al., 2003), promoting neural pathway reconstruction after spinal cord injury and stroke (Jiang et al., 2017; Tang et al., 2017), and providing alternative cell sources for glial cell injury-related diseases like multiple sclerosis (Encinas and Fitzsimons, 2017; Mouhieddine et al., 2014). However, current research faces multiple bottlenecks: (1) Challenges in cell identification and heterogeneity: NSCs lack specific molecular markers, making it difficult to precisely isolate quiescent and activated subpopulations, and their differentiation potential often shifts after *in vitro* expansion (e.g., reduced neuronal differentiation capacity) (Đ_._ăng et al., 2019; Encinas and Fitzsimons, 2017; Nam et al., 2015); (2) Unclear mechanisms of differentiation control and microenvironmental interactions: the regulation of differentiation by growth factor combinations is stochastic, and inflammatory microenvironments significantly inhibit transplanted cell survival (Deokate et al., 2024; Encinas and Fitzsimons, 2017; Mouhieddine et al., 2014); (3) Low efficiency of clinical translation: human NSC samples are scarce, animal models fail to fully replicate human disease phenotypes, and transplanted cell survival rates are low (<10%) with side effects such as dyskinesia (Deokate et al., 2024; Nam et al., 2015; Olanow et al., 2003); (4) Barriers to multi-scale data integration: there is a lack of unified analytical frameworks for cross-omics, imaging, and electrophysiological data, and reliance on manual processing leads to low efficiency and high bias (Pathan et al., 2022; Ramakrishna et al., 2020). In recent years, the rapid development of artificial intelligence (AI) technologies has provided new ideas and tools to address these challenges. In this review, we will focus on the current applications of AI-related technologies in NSCs research.

## 2 The paradigm shift in NSCs basic research driven by AI

**FIGURE 1:**
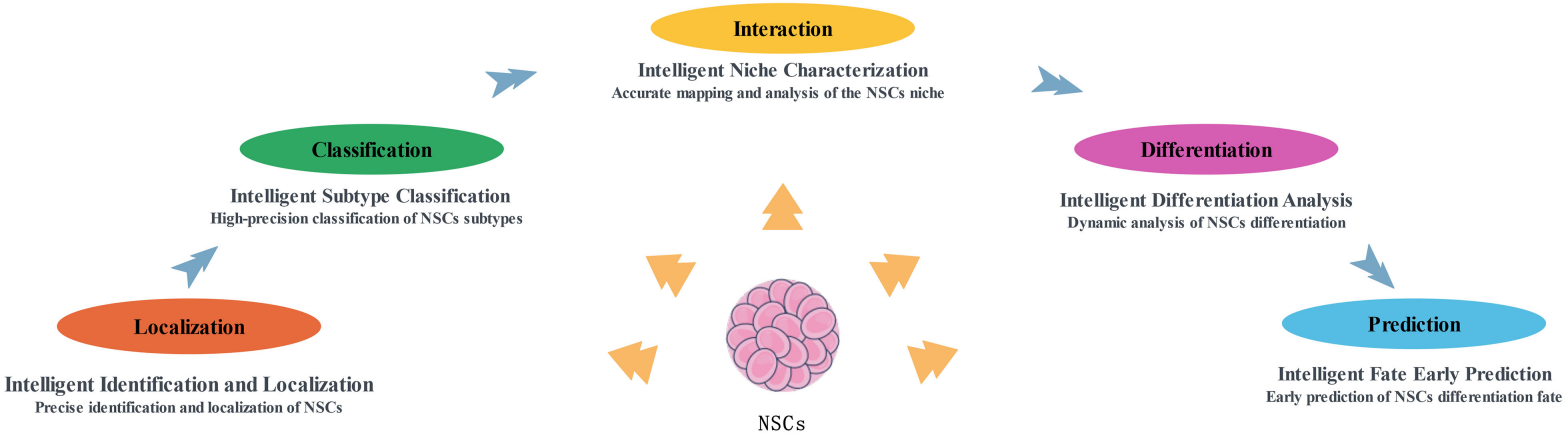
The main application directions of artificial intelligence in NSCs research at present. NSCs, neural stem cells.

Currently, artificial intelligence technology in neural stem cell research is primarily focused on intelligent identification and localization of neural stem cells, precise determination of their subtypes, analysis of the microenvironment (niche) surrounding neural stem cells, elucidation of the dynamic differentiation processes, and early-stage prediction of their fate decisions. These studies cover the core areas of neural stem cell research and have advanced the in-depth understanding of their biological characteristics and functional mechanisms (Figure 1).

### 2.1 AI-enabled intelligent recognition techniques for NSCs

Neural stem cells play a critical role in nervous system development, regeneration, and repair, making their precise localization and identification crucial. Traditional cell identification methods are often limited by the scarcity of cells and significant individual variability, posing challenges to the accurate localization of NSCs. With the advancement of AI technologies, especially the application of machine learning (ML) algorithms, new solutions have emerged for the automated recognition of NSCs.

Dumitru et al. (2025) in their analysis of adult hippocampal samples, first performed preliminary screening of cells using antibody labeling of the proliferation marker Ki67, followed by deep recognition and classification of cell features through ML algorithms, successfully identifying proliferative neural progenitor cells. This technological breakthrough overcame the limitations of traditional methods and effectively addressed the challenges of precise localization caused by low cell numbers and high individual variability (Dumitru et al., 2025). Hailstone et al. (2020) employed supervised ML algorithms to develop CytoCensus, which, trained by user clicks on cell centers, enables automatic identification, counting, and quantification of division behaviors of NSCs and their derivatives without relying on specific cell markers or custom programming. Mateo et al. (2015) used ML methods to identify gene regulatory features associated with NSCs self-renewal, differentiation, and quiescent states. Ogi et al. (2019) modeled hyperspectral data using ML to achieve non-destructive classification of neurons and glial cells, providing a novel approach for precise cell type identification.

In the study of early neurogenesis in the zebrafish forebrain (telencephalon), Stringer et al. (2021) used the ML-based 3D cell segmentation algorithm Cellpose, to quantify the total number of progenitor cells and neurons during the first phase of telencephalon development, particularly focusing on how NSCs transition from proliferative divisions to neurogenic divisions (Casas Gimeno et al., 2023). Smith et al. (2017) applied the CoNTexT ML algorithm to exon expression array data to determine the regional characteristics and developmental maturity of NSCs in both 2D and 3D cultures.

The application of these AI-related technologies provides strong support for the intelligent identification of NSCs and lays the foundational groundwork for further NSC research. Importantly, these approaches have transformed our understanding by enabling quantitative analysis of NSC behavior within their native tissue context. Nevertheless, the effectiveness of these tools can be influenced by factors such as the requirement for high-quality annotated data for training (Hailstone et al., 2020; Stringer et al., 2021) and potential sensitivity to complex tissue architectures and imaging conditions, which may impact generalizability across diverse experimental settings.

### 2.2 Deep learning-driven in-depth analysis of NSCs subtypes

Neural stem cells are crucial components in nervous system development and regeneration. Based on developmental stages, anatomical locations, and molecular characteristics, NSCs can be classified into different subtypes, which exhibit significant differences in differentiation capacity and function. Different NSCs subpopulations express specific markers such as Sox2, Pax6, and Nestin, but their expression levels show notable heterogeneity. For example, cells with high Sox2 expression tend to maintain a stem cell state, whereas cells with high Pax6 expression are more prone to differentiate into neurons. This difference in differentiation potential provides important clues for understanding neural development and regeneration (Galiakberova and Dashinimaev, 2020). Functionally, NSCs can be divided into quiescent NSCs (qNSCs) and activated NSCs (aNSCs). qNSCs are in the G0 phase, primarily rely on lipid oxidation for metabolism, and highly express adhesion molecules (such as genes related to the Notch pathway), thereby maintaining their stem cell characteristics (Ding et al., 2020; Yu et al., 2024). In contrast, aNSCs shift their metabolism toward mitochondrial oxidation, can initiate neurogenesis, and can be further subdivided into pre-activated and proliferative states (Dimitrakopoulos et al., 2022; Ding et al., 2020). Effectively distinguishing between quiescent and activated NSC subtypes helps deepen the understanding of neurogenesis and the dynamic regulatory mechanisms of stem cells, thereby promoting research into neural regeneration and repair.

Dulken et al. performed an in-depth analysis of NSC subtypes using Monocle ordering and found that aNSCs can be further subdivided into specific subgroups, including aNSC-early, aNSC-mid, and aNSC-late, each characterized by distinct surface markers and key intracellular regulators. This finding provides a new perspective on the functional diversity of NSCs (Dulken et al., 2017). Xie et al. developed a tool named scAIDE, an unsupervised deep learning clustering framework for single-cell RNA sequencing (scRNA-seq) data, aimed at identifying rare and potential cell types. To overcome the high noise in the data, scAIDE first combines an autoencoder imputation network with an anchor-based embedding network (AIDE) to learn effective data representations, then applies random projection hashing combined with a k-means algorithm to detect rare cell types. In a large dataset containing 1.3 million neural cells, scAIDE successfully identified 64 clusters mapped to 19 potential cell types, including NSCs and progenitor cells. Moreover, scAIDE was able to identify three distinct developmental trajectories of NSCs, providing important insights into the differentiation process of NSCs (Xie et al., 2020). Chen et al. (2018) used Monte Carlo Feature Selection (MCFS) to analyze gene expression data from three NSC subtypes—quiescent NSCs (qNSCs), activated NSCs (aNSCs), and neural progenitor cells (NPCs)—to identify their molecular characteristics and classification markers. After selecting key gene expression features with the MCFS algorithm, they combined these with a Support Vector Machine (SVM) to construct a predictive model, which was evaluated using ten-fold cross-validation. The model achieved a high classification accuracy with a Matthews correlation coefficient (MCC) of 0.918. Additionally, the MCFS algorithm generated classification rules distinguishing the three subtypes, revealing subtype-specific gene expression patterns and their dynamic changes during NSCs lineage differentiation (Chen et al., 2018). Chen et al. (2024) employed ML-based algorithms to construct a consensus brain cell atlas with annotated cell types, demonstrating the presence of neural progenitor subpopulations in the brain. The application of these AI technologies has not only enabled high-precision classification of NSCs subtypes but also elucidated their gene expression features, providing new insights into the molecular basis of neural development. These methodologies have significantly refined our conceptual framework by revealing a continuum of NSC activation states (Dulken et al., 2017) and complex developmental trajectories25, thereby enhancing the understanding of NSC heterogeneity in neural development. A key consideration in leveraging these powerful tools is addressing challenges such as the inherent noise and batch effects in single-cell data that can complicate the discernment of true biological variation (Xie et al., 2020), and the recognition that trajectory inference models might oversimplify the dynamic, potentially non-linear nature of NSC state transitions *in vivo*.

### 2.3 ML-driven deconstruction of the NSCs spatial microenvironment

The function and fate of NSCs are significantly influenced by their microenvironment, and understanding the spatial microenvironment these cells reside in is crucial for revealing their biological characteristics and regenerative capacity. Cell interactions, signaling, and spatial distribution within the microenvironment play key roles in NSCs self-renewal and differentiation. With the advancement of ML technologies, researchers are now able to more precisely map and analyze NSC behaviors within complex microenvironments.

Marymonchyk et al. (2025) mapped an accurate spatiotemporal atlas of NSCs functional responses induced by multiple niche cell types within the subventricular zone NSCs niche of the adult mouse brain and utilized ML to predict interactions between NSCs and specific niche cell types. This study provides important data support for understanding the dynamic behavior of NSCs within their microenvironment (Marymonchyk et al., 2025). Sun et al. (2025) developed “spatial ageing clocks,” a ML-based tool trained on a single-cell resolution spatial transcriptomic atlas of 4.2 million cells in the mouse brain, covering 20 age groups and two regenerative interventions. This tool identifies spatial and cell type-specific transcriptomic features of aging and regeneration, revealing that NSCs exhibit a significant “pro-rejuvenating proximity effect” on neighboring cells—meaning their spatial positioning can markedly suppress aging phenotypes in surrounding cells. This effect could not be captured by traditional non-spatial analyses and represents the first quantification of NSCs-mediated regenerative regulation within tissue microenvironments, highlighting the critical value of AI in deciphering spatial functions of rare cell types (Sun et al., 2025). Spatial Genomic Analysis (SGA) is a quantitative single-cell transcriptome analysis method that uses single-molecule imaging of up to one hundred gene transcripts, applicable to various *in situ* applications, and capable of identifying subpopulations within the dorsal neural tube neural crest niche (Lignell and Kerosuo, 2002). Lignell et al. (2017) developed a ML-based image analysis pipeline to identify single-cell contours from 3D image stacks and defined a neural crest stem cell niche centered around the dorsal midline.

The application of these ML techniques enables a clearer depiction of the spatial environment of NSCs, revealing their interactions with surrounding cells and their effects on tissue regeneration and aging and providing an important theoretical foundation and technical support for future precise interventions targeting NSCs functions. A profound insight from this line of research is the demonstration of non-cell-autonomous functions of NSCs, as evidenced by the “pro-rejuvenating proximity effect” (Sun et al., 2025), which underscores their role in modulating tissue homeostasis beyond cell-autonomous activities. When applying these spatial analysis techniques, it is important to consider current limitations, including the resolution and molecular capture efficiency of spatial technologies that may affect the delineation of fine-scale interactions (Sun et al., 2025), and the computational challenges associated with integrating heterogeneous spatial datasets.

### 2.4 ML-driven deconstruction of neural differentiation dynamics

Neural differentiation is fundamental to nervous system development and regeneration. A deep understanding of its dynamic regulatory mechanisms is crucial for elucidating the pathology of neurodevelopmental diseases and developing regenerative medical therapies. However, neural differentiation efficiency varies significantly among different cell lines, and its regulatory mechanisms are complex and diverse, making comprehensive analysis challenging with traditional experimental methods. Therefore, employing advanced ML approaches to systematically deconstruct neural differentiation dynamics has become an important means to advance research in this field.

Yu et al. (2023) applied the ML pipeline CellBiAge to analyze single-cell transcriptomic data, accurately classifying the age of individual cells in the mouse brain and successfully capturing the promoting effect of exercise on the regenerative capacity of proliferative NSCs in the subventricular zone (SVZ). Guerin et al. (2025) developed a ML model to capture and predict the complex relationships among 5-methylcytosine (5-mC), 5-hydroxymethylcytosine (5-hmC), and chromatin accessibility (ChrAcc), enabling prediction of past, present, and future chromatin accessibility states and thereby elucidating neural progenitor differentiation processes. Sekiya et al. (2022) employed a ML–based non-linear feature selection method, HSIC Lasso (Hilbert-Schmidt Independence Criterion Lasso), to analyze genome-wide DNA methylation data of 32 human induced pluripotent stem cell (hiPSC) lines in the undifferentiated state along with their neural differentiation efficiencies. They successfully identified 62 CpG sites significantly associated with neural differentiation efficiency from the entire epigenome, establishing for the first time a predictive model of neural differentiation capacity based on epigenetic features of undifferentiated hiPSCs. This provides key biomarkers for efficiently screening cell lines suitable for neural differentiation studies (Sekiya et al., 2022).

These ML techniques demonstrate powerful data mining and predictive capabilities in deciphering neural differentiation dynamics. They not only reveal critical epigenetic markers associated with differentiation efficiency but also achieve precise modeling of cellular age and chromatin states, offering essential tools and theoretical foundations for understanding neural development mechanisms and optimizing stem cell differentiation strategies. The significant advancement here lies in the ability to integrate multi-scale data to link pre-existing molecular signatures, such as epigenetic states in undifferentiated cells (Sekiya et al., 2022), with their future differentiation potential, thereby offering a predictive, systems-level perspective on NSC fate determination. To fully leverage these models, awareness of their limitations is essential, including their dependence on high-quality, longitudinally sampled data that can be resource-intensive to acquire (Sekiya et al., 2022; Yu et al., 2023), and the fact that they often yield correlative predictions that necessitate further experimental validation to establish causative mechanisms.

### 2.5 Early non-invasive prediction of differentiation fate mediated by deep learning

Neural stem cells differentiation has long faced several key challenges. First, the differentiation process is highly complex and uncontrollable: NSCs differentiate into neurons, astrocytes, or oligodendrocytes through dynamic gene networks and interactions with microenvironmental factors. Traditional experiments require 5–7 days to verify results via immunofluorescence or Western blot, which is time-consuming and inefficient (Liu et al., 2013; Wang and Barres, 2000; Wang et al., 2012). Second, early morphological clues during differentiation (<48 h), such as cell body contraction and nuclear displacement, though indicative of fate, are difficult to quantify precisely by manual observation (Zhu et al., 2021). Additionally, clinical translation is hindered by the randomness of differentiation direction, leading to unstable neuron proportions after transplantation (Zhu et al., 2021). Therefore, early prediction of NSC differentiation direction could overcome time window limitations, accelerate cell therapy development, and provide a basis for personalized neural regeneration.

Zhu et al. (2021) developed a model based on the Xception convolutional neural network to detect very subtle morphological changes in bright-field single-cell images. This approach enables highly accurate prediction of neuron/glial fate within only 0.5–1 day after differentiation initiation, much faster than the traditional 5 days using label-free bright-field images. They also introduced class activation mapping (CAM) to localize key decision regions at the cell edges and internal details, providing morphological clues for differentiation mechanisms (Zhu et al., 2021). Forster applied deep learning to characterize developmental changes in neural progenitor cells by accurately identifying and quantifying different cell types (including radial glia, neurons, astrocytes, and oligodendrocytes) in migration regions, demonstrating robustness against typical confounding factors (Förster et al., 2022). Geng et al. (2021) used Raman spectroscopy to obtain biochemical feature data during NSC differentiation and applied ML for data processing and model building to distinguish NSCs from neurons. This enabled real-time, accurate tracking of NSC differentiation at the single-cell level, offering an efficient strategy for clinical applications (Geng et al., 2021). Hanafusa et al. (2023) employed the Google Cloud AutoML Vision platform to develop a ML model based on calcium spark waveform images, analyzing ATP-triggered calcium responses in human induced pluripotent stem cell-derived NSCs (iNSCs) and achieving high-precision classification of iNSC calcium response waveforms. This capability marks a paradigm shift toward recognizing early biomechanical and morphological changes as determinative events in fate commitment (Zhu et al., 2021). A crucial aspect of employing these advanced prediction tools involves navigating their limitations, such as the “black box” nature of deep learning models where the underlying biological mechanisms can remain elusive despite techniques like CAM (Zhu et al., 2021), and potential constraints in model generalizability across different cell lines and culture conditions.

These deep learning technologies (Table 1) enable rapid, non-invasive prediction of NSC differentiation fate, significantly shortening traditional detection times, achieving high-precision automated identification, and advancing neural differentiation research and clinical applications.

**TABLE 1:** Summary of AI Technologies applied in NSC research.

AI technology	Key applications	Biological insight gained	Limitations / challenges	References
ML-based recognition	Automated NSC counting, localization, and division tracking	Quantitative analysis of NSC proliferation dynamics and spatial distribution in tissues	Sensitive to image quality; requires expert annotation; performance varies across tissue types	Casas Gimeno et al., 2023; Dumitru et al., 2025; Hailstone et al., 2020
DL-based subtype analysis	Identification of NSC subtypes and developmental trajectories	Revealed continuum of NSC states and molecular heterogeneity underlying differentiation capacity	Requires large datasets; computational intensity; batch effect sensitivity; may oversimplify dynamics	Chen et al., 2018; Dulken et al., 2017; Xie et al., 2020
ML for spatial analysis	Mapping NSC-niche interactions; analyzing spatial transcriptomics	Uncovered non-cell-autonomous mechanisms (e.g., pro-rejuvenating effect of NSCs)	Computationally intensive; limited by resolution and coverage of spatial technologies	Marymonchyk et al., 2025; Sun et al., 2025
ML for differentiation dynamics	Modeling epigenetic regulation; predicting differentiation efficiency	Linked pre-programmed epigenetic patterns and external stimuli to NSC fate	Limited by sample size and temporal resolution of data; predictions are often correlative	Sekiya et al., 2022; Yu et al., 2023
DL for fate prediction	Early morphology-based fate classification	Demonstrated that early biomechanical cues precede molecular commitment in differentiation	Limited generalizability across conditions; lack of mechanistic explanation; “black box” problem	Zhu et al., 2021

## 3 Key AI technologies not yet applied to NSC research

**FIGURE 2:**
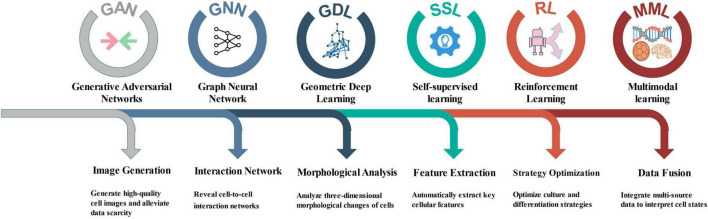
Key AI technologies not yet applied to NSC research.

With the rapid development of AI technologies, many techniques successfully applied in fields outside of NSCs research show great potential for advancing studies in this area. The following are several key AI technologies that have not yet been widely applied to NSCs research, along with their potential applications (Figure 2).

Generative Adversarial Networks (GAN) are deep learning models used to generate new data by producing synthetic samples that resemble real data during training. Aida et al. (2020) utilized conditional GANs (CGAN) to study the segmentation of cancer stem cells in phase-contrast imaging. Similarly, in NSC research, GANs can be employed to generate high-quality cell images, assisting researchers in analysis when data are scarce. Moreover, GAN can be used for data augmentation by generating cell images with various transformations, thereby improving the robustness and accuracy of models. This approach could be particularly valuable when integrated with the early fate prediction methods discussed in section 2.5 “Early non-invasive prediction of differentiation fate mediated by deep learning,” potentially enabling researchers to explore causal relationships between specific morphological features and differentiation outcomes, thereby moving beyond correlative analyses to testable causal hypotheses.

Graph Neural Networks (GNN) are deep learning models designed to handle graph-structured data, effectively capturing relationships and structural information among nodes. Yadalam et al. (2024) found that GNN outperform traditional ML methods in predicting drug-gene interactions within the RTK-VEGF protein family during periodontal regeneration. Likewise, in NSC research, GNN can be applied to analyze cell–cell interaction networks, revealing the dynamic behaviors of NSCs within their microenvironment. For example, GNN can help identify signaling pathways between different cell types, thereby improving understanding of how NSCs self-renew and differentiate in specific microenvironments. By constructing dynamic models of the NSC niche interactions described in section “2.3 ML-driven deconstruction of the NSCs spatial microenvironment,” GNNs could provide insights into how network perturbations propagate through the cellular microenvironment, potentially identifying critical regulatory nodes that control NSC fate decisions at a systems level.

Geometric Deep Learning (GDL) is a deep learning approach for processing non-Euclidean data such as point clouds and meshes, effectively capturing complex shapes and structural information. The three-dimensional (3D) morphology of cells, arising from intricate cell–environment interactions, serves as an indicator of cell state and function. De Vries et al. (2025) combined GDL with attention-based multiple instance learning pipelines to characterize the 3D shapes of cells and nuclei. In NSC research, GDL can similarly be used to analyze morphological changes of cells and identify features at different developmental stages. When applied to complement the subtype analysis in section “2.2 Deep learning-driven in-depth analysis of NSCs subtypes,” GDL could help establish meaningful correlations between cellular morphology and molecular identity, potentially enabling image-based prediction of NSC states and functional potential.

Self-supervised learning (SSL) is a technique that automatically learns effective feature representations from unlabeled data. This approach has demonstrated strong performance in fields such as image processing and natural language processing and is especially suitable for biomedical scenarios where labeled data is scarce. Ramesh et al. (2024) developed a deep learning model based on SSL for automated diagnosis and precise classification of neuroblastoma. In NSCs research, SSL can similarly leverage large amounts of unannotated cell images or single-cell sequencing data to automatically extract key features such as cell morphology and gene expression, thereby reducing reliance on expensive and time-consuming manual annotation and improving the model’s generalization ability and robustness. Applied to the differentiation dynamics discussed in section “2.4 ML-driven deconstruction of neural differentiation dynamics,” SSL could facilitate the discovery of novel differentiation trajectories or intermediate states that may be overlooked by supervised approaches, thereby providing a more comprehensive understanding of NSC lineage commitment.

Reinforcement Learning (RL) in cell biology research can achieve precise regulation and functional enhancement of cell behavior through dynamic interaction between agents and the environment, continuously optimizing experimental strategies based on feedback signals. Wang et al. (2022) used deep RL to infer intercellular interactions and collective cell behavior in tissue morphogenesis from 3D delayed images, in order to examine cell migration. In NSCs research, RL can be used to optimize cell culture conditions, differentiation induction schemes, and drug screening processes. When combined with the early prediction capabilities described in section “2.5 Early non-invasive prediction of differentiation fate mediated by deep learning,” RL could enable adaptive optimization of differentiation protocols in real-time, potentially uncovering optimal temporal sequences of cues that maximize differentiation efficiency toward specific lineages.

Multimodal learning (MML) aims to integrate information from different types of data—such as images, gene expression, spatial transcriptomics, and electron microscopy images—to construct more comprehensive cellular representation models. Khodaee et al. (2025) developed a MML model that can explore genotype-phenotype relationships in human transcriptomics at the cellular level. NSCs research also involves various heterogeneous data types, and traditional single-modality analyses struggle to reveal the complex cellular states and functional relationships. Through MML, morphological, molecular, and spatial information can be integrated to deeply investigate NSCs developmental trajectories, microenvironmental influences, and differentiation fates. This approach could unify the spatial context from section “2.3 ML-driven deconstruction of the NSCs spatial microenvironment” with molecular profiles from section “2.2 Deep learning-driven in-depth analysis of NSCs subtypes” and morphological data from section “2.5 Early non-invasive prediction of differentiation fate mediated by deep learning,” creating unified models that bridge genetic information with functional outcomes across multiple biological scales.

This summary highlights the potential applications, biological insights, and synergistic opportunities with established methods presented by these emerging AI technologies (Table 2). Integrating these tools with current approaches has the potential to overcome prevalent challenges in the field, such as data scarcity and limited model interpretability, thereby shedding new light on neural stem cell biology. Importantly, the path to successful application involves addressing key hurdles like data quality and algorithm robustness, which are discussed in the subsequent section.

**TABLE 2:** Potential of emerging AI technologies in NSC research.

AI technology	Key applications in NSC research	Expected biological insights	Integration potential with section “2 The paradigm shift in NSCs basic research driven by AI” methods
GANs	Data augmentation; Synthetic image generation	Causal relationships between morphology and cell fate	With section “2.5 Early non-invasive prediction of differentiation fate mediated by deep learning”: Testing causality in fate determination
GNNs	Dynamic modeling of cell-cell interactions	Systems-level understanding of niche regulation	With section “2.3 ML-driven deconstruction of the NSCs spatial microenvironment”: Predicting niche perturbation effects
GDL	3D morphological analysis	Linking cell shape to functional potency	With section “2.2 Deep learning-driven in-depth analysis of NSCs subtypes”: Defining morpho-molecular subtypes
SSL	Unsupervised feature learning	Discovering novel NSC states and trajectories	With section “2.4 ML-driven deconstruction of neural differentiation dynamics”: Identifying differentiation heterogeneity
RL	Optimization of culture conditions	Decoding temporal rules of differentiation	With section “2.5 Early non-invasive prediction of differentiation fate mediated by deep learning”: Dynamic protocol optimization
MML	Multi-scale data integration	Unified models connecting genotype to phenotype	With sections “2.2 Deep learning-driven in-depth analysis of NSCs subtypes”/“2.3 ML-driven deconstruction of the NSCs spatial microenvironment”/“2.5 Early non-invasive prediction of differentiation fate mediated by deep learning”: Cross-modal data correlation

## 4 Challenges faced: data quality, model robustness, and interpretability

Although AI technology shows great potential in NSCs research, its application still faces multiple challenges, particularly in terms of data quality, model robustness, and interpretability. First, data quality and standardization issues are key bottlenecks limiting AI model performance. NSCs experimental data often come from various high-throughput technologies such as single-cell RNA sequencing, proteomics, and microscopy imaging. These data typically suffer from high noise levels, significant batch effects, limited sample sizes, and strong heterogeneity. Noise and sequencing errors can introduce bias during model training, affecting prediction accuracy and stability. Moreover, inconsistent data standards across different laboratories and platforms further complicate data integration and model generalization. Therefore, effective preprocessing, standardization, and quality control of NSCs data are fundamental to improving AI model reliability.

Second, the robustness of models faces significant challenges. The complexity and diversity of NSCs data cause model performance to degrade when confronted with noise, missing data, or distribution shifts. Existing models are often sensitive to data perturbations and lack sufficient generalization ability, making it difficult to handle data variations arising from different experimental conditions or clinical settings. Furthermore, models tend to overfit or produce unstable predictions when dealing with small sample sizes, high-dimensional, and heterogeneous data. To improve model robustness, it is necessary to design more effective regularization strategies, employ reinforcement learning methods, and utilize adversarial training techniques to enhance model stability and generalization across various complex environments.

Finally, the “black-box” nature of AI models limits their application in both clinical and basic research. Many deep learning models have complex structures and opaque decision-making processes, making it difficult for researchers to understand the reasoning logic and key driving factors behind their predictions. This not only affects the credibility of scientific discoveries but also hinders the adoption of AI technologies in clinical diagnosis and treatment. As NSCs research moves toward clinical translation, model interpretability and transparency become increasingly important. Developing explainable AI (XAI) methods will help enhance trust in research findings, facilitate interdisciplinary collaboration, and promote clinical adoption.

Looking ahead, advancing AI applications in the NSCs field hinges on building high-quality, standardized multimodal data platforms to ensure data reliability and consistency. At the same time, integrating biological knowledge into hybrid models is necessary to enhance model robustness and biological interpretability. Promoting innovations in explainable AI (XAI) technologies will improve model transparency and credibility, fostering closer integration between basic research and clinical applications, while strengthening interdisciplinary collaboration and data sharing.

Although current applications of AI technologies in NSC research remain relatively limited, these emerging techniques are expected to play increasingly significant roles as they continue to develop and mature. By integrating advanced technologies such as GNN, GAN, and GDL et.al, researchers will be able to achieve a more comprehensive understanding of the biological characteristics, differentiation mechanisms, and regenerative potential of NSCs. This not only provides new tools for basic research but also opens up new possibilities for clinical applications and personalized therapies.
